# The Role of a Spinning Top Urethra in the Development of Pyosalpinx in a Precoital Female

**DOI:** 10.7759/cureus.27099

**Published:** 2022-07-21

**Authors:** Ted J Andrews, Mark Hicar, Shamim Islam

**Affiliations:** 1 Pediatrics, University at Buffalo, Jacobs School of Medicine and Biomedical Sciences, Buffalo, USA

**Keywords:** precoital females, pyosalpinx, pediatric infectious disease, pediatric ob/gyn, spinning top urethra

## Abstract

Pelvic inflammatory disease (PID) is commonly related to sexual intercourse in postpubescent females. Inflammation of the fallopian tubes (pyosalpinx) is a common complication. We describe a case of anatomic spinning top-shaped urethra that led lead to complicated recurrent severe abdominal infections in an 11-year-old precoital female. This case highlights that prepubescent females can have pyosalpinx and pelvic inflammatory disease and anatomic contributions to these presentations should be considered.

## Introduction

Pelvic inflammatory disease (PID) is commonly related to sexual intercourse in postpubescent females. Inflammation of the fallopian tubes (pyosalpinx) is a common complication [1]. We describe a case of anatomic spinning top-shaped urethra that led lead to complicated recurrent severe abdominal infections in an 11-year-old precoital female. This case highlights possible anatomic contributions to pyosalpinx and pelvic inflammatory disease in prepubescent females.

## Case presentation

An 11-year-old Caucasian premenarchal female was brought to the emergency department (ED) by her parents with a complaint of worsening right lower quadrant pain noticed when she awoke that morning. It began as four out of 10 pain and escalated to eight out of 10 over the course of hours. She had no associated prior fever, nausea, vomiting, constipation, hematochezia, diarrhea, weight loss, hematuria, dysuria, urgency, or frequency. She had no significant travel, her vaccinations were up-to-date, and she denied any sick contacts. She had a remote history of recurrent urinary tract infections (UTIs), without use of prophylactic antibiotics or recurrences for over five years. She denied any past or recent history of sexual activity.

**Figure 1:**
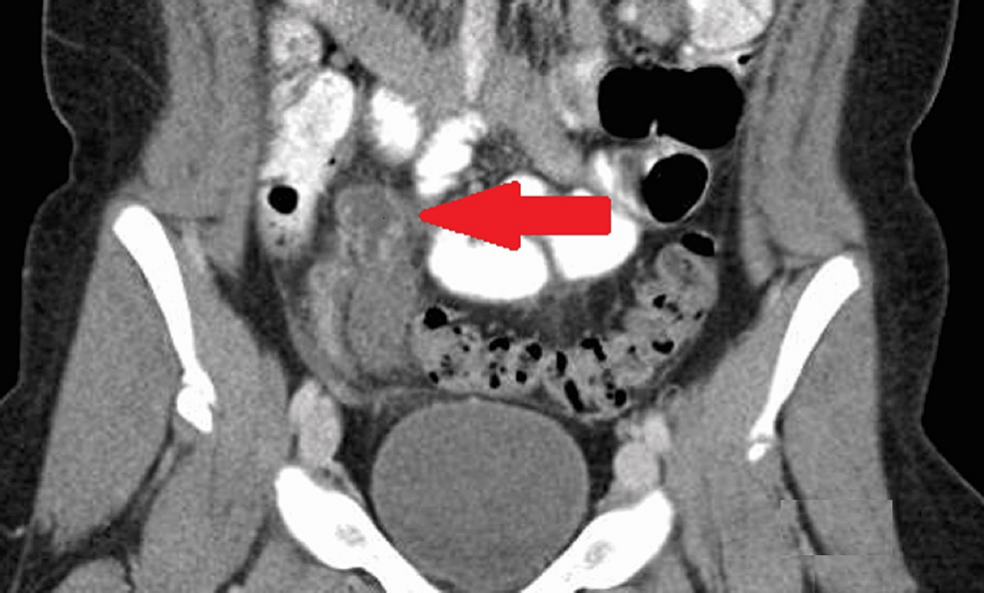
CT abdomen showing right hydrosalpinx

On examination, she was febrile to 38.9°C, with heart rate of 110 beats per minute, a blood pressure of 92/54, a respiratory rate of 20 breaths per minute, and an oxygen saturation of 99% on room air. BMI was > 97th percentile. She was non-toxic in appearance, and her abdomen was soft with moderate right lower quadrant tenderness, minimal guarding, and no rebound tenderness. The remainder of her physical examination was within normal limits. Her white blood cell (WBC) count was 20.5 × 10^9^/L with the following differential: 76% neutrophils, 12% bands, 5% lymphocytes, and 7% monocytes. She had otherwise normal laboratory investigations, including liver function tests, with only a mild metabolic acidosis noted. Pelvic ultrasound with Doppler showed a hypoechoic tubular structure abutting the ovary with surrounding area of increased echogenicity. Figure 1 shows computed tomography (CT) with IV contrast of the abdomen and pelvis revealed right hydrosalpinx with surrounding fat stranding. With gynecological and pediatric surgery team involvement, the patient had a laparoscopic dilatation of right fallopian tube with pelvic irrigation and prophylactic removal of a non-inflamed retrocecal appendix. The infectious disease service was consulted, and the patient was admitted for antibiotic therapy.

The intraabdominal wound culture grew a pansensitive *Escherichia coli* while the urine culture had no growth at 1,000 CFU. Additional testing for gonorrhea, chlamydia, and trichomonas was negative. A genital culture grew *Streptococcus agalactiae*. On discharge, the patient was changed to oral levofloxacin and metronidazole to complete a 14-day course. On follow-up with gynecology and infectious diseases, her symptoms were completely resolved, and no further testing or prophylactic antibiotic regimens were recommended.

**Figure 2:**
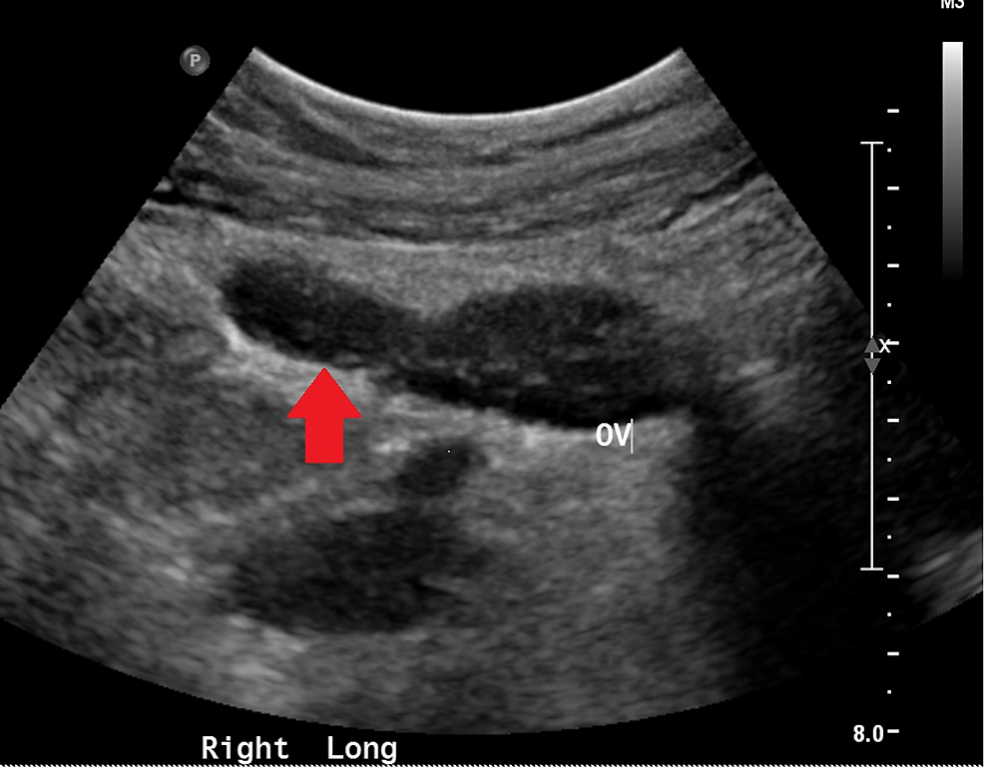
Ultrasound (US) showing bilateral dilated thick-walled fallopian tubes containing debris

Seven months later, the patient presented to her primary medical doctor (PMD) for left lower quadrant pain for two days with associated fever of 38.3°C. An ultrasound showed bilateral dilated thick-walled fallopian tubes containing debris and her PMD prescribed levofloxacin (Figure 2).

**Figure 3:**
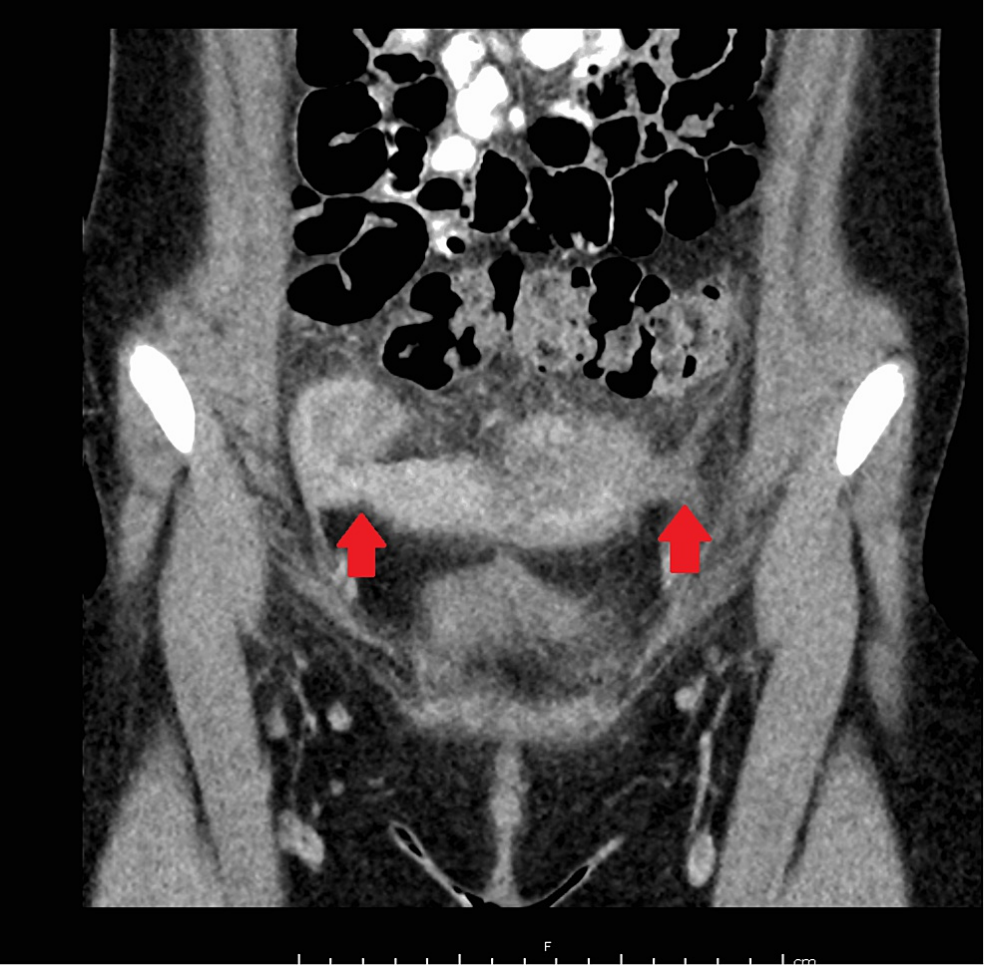
CT abdomen showing bilateral hydrosalpinx right greater than left

The next day, she was brought to the ED with worsening left lower quadrant pain. She again denied nausea, vomiting, constipation, hematochezia, diarrhea, weight loss, hematuria, dysuria, urgency, and frequency. On examination, she was febrile at 38.7°C, heart rate was 99 beats per minute, a blood pressure of 117/82 mmHg, a respiratory rate of 20 breaths per minute, and oxygen saturation of 99%. Laboratory examination included a WBC of 18.5 × 10^9^/L with 75.4% segmented neutrophils and c-reactive protein (CRP) of 182.63 mg/L. Urinalysis showed 2+ leukocyte esterase and 26-100 leukocytes per high-powered field. Urine culture was negative and suspected to be secondary to prior antibiotic use. The patient was again admitted to the floor with gynecologic consultation, and meropenem was initiated. A CT scan with contrast was obtained to investigate a possible anatomic fistulous tract contributing to recurrent infections. Figure 3 shows the CT which was notable for bilateral tubular-filled structures consistent with dilated fallopian tubes but no evidence of fistula. At this time, the patient’s mother relayed that the patient frequently had stool-soiled underwear, poor hygiene, and poor toileting habits including concern of wiping back to front. The patient completed a 14-day course of the meropenem. 

Four months later, the patient presented to the ED with non-radiating, stabbing right lower quadrant abdominal pain, 6/10 in intensity. She reported that the day prior she had played in four different lacrosse games, but denied any trauma or injury. The patient also denied fever, dysuria, hematuria, and vaginal discharge. Between these last two ED visits, consultation by a pediatric gastroenterologist revealed the patient was vitamin D deficient and had an IgA deficiency, but celiac disease and inflammatory bowel disease (IBD) were ruled out. Mother reported that she had menarche, with a period lasting a single day roughly one month prior to this presentation. The patient again denied any sexual activity. Notable laboratory values included WBC 10.2 × 10^9^/L with 65% neutrophils and urinalysis with 1+ leukocyte esterase and positive nitrites. The patient was admitted and started on levofloxacin 500 mg qD and metronidazole 500 mg q12.

**Figure 4:**
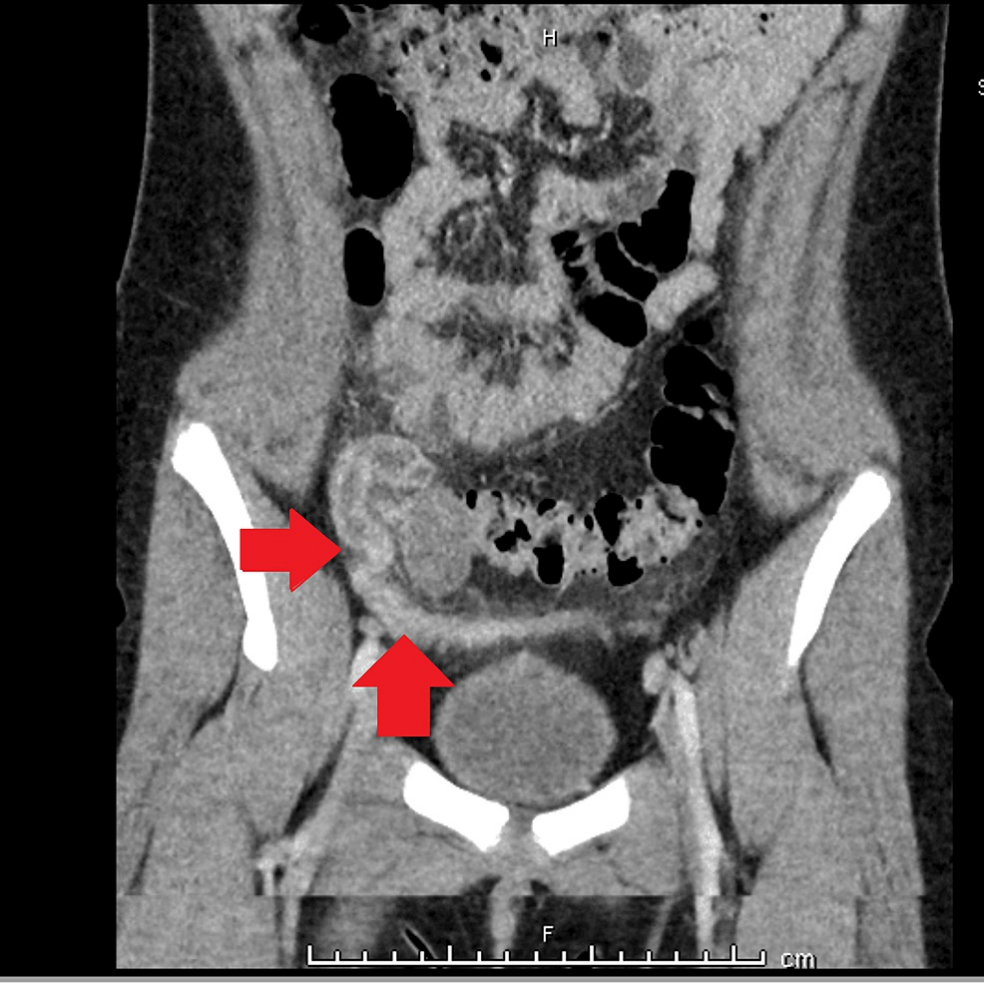
CT abdomen with right fallopian tube with surrounding inflammatory change

A pelvic ultrasound was not definitive. Gynecologic consultation recommended pelvic CT which showed mild dilatation of the right fallopian tube with surrounding inflammatory change consistent with pyosalpinx or PID (Figure 4).

Urine culture showed greater than 100,000 CFU/ml of *E. coli* and levofloxacin was continued. A laparoscopic assisted gynecologic exam under anesthesia was performed. The patient’s mons showed mild erythema in an oval pattern around the introitus and labia majora. Yellow smegma was noted in the vulvar folds. Her urethra was in close proximity to her vaginal opening with 1 cm separation, a normal finding. Her hymen was normal, without lesion. A moderate amount of clear discharge with mucous was noted within the vagina. An aspirate of that fluid grew rare lactobacillus. A vaginal culture grew rare beta-hemolytic streptococcus B while an anaerobic vaginal culture grew anaerobic Gram-negative bacilli. Rectal examination did not reveal any lesions or irregularities between rectum and vaginal wall. There was no evidence of a fistula. Bimanual exam revealed a normal, small uterus and no palpable masses. The patient remained afebrile and her abdominal pain lessened to 1/10. She was discharged home to complete a total 14-day course of levofloxacin.

**Figure 5:**
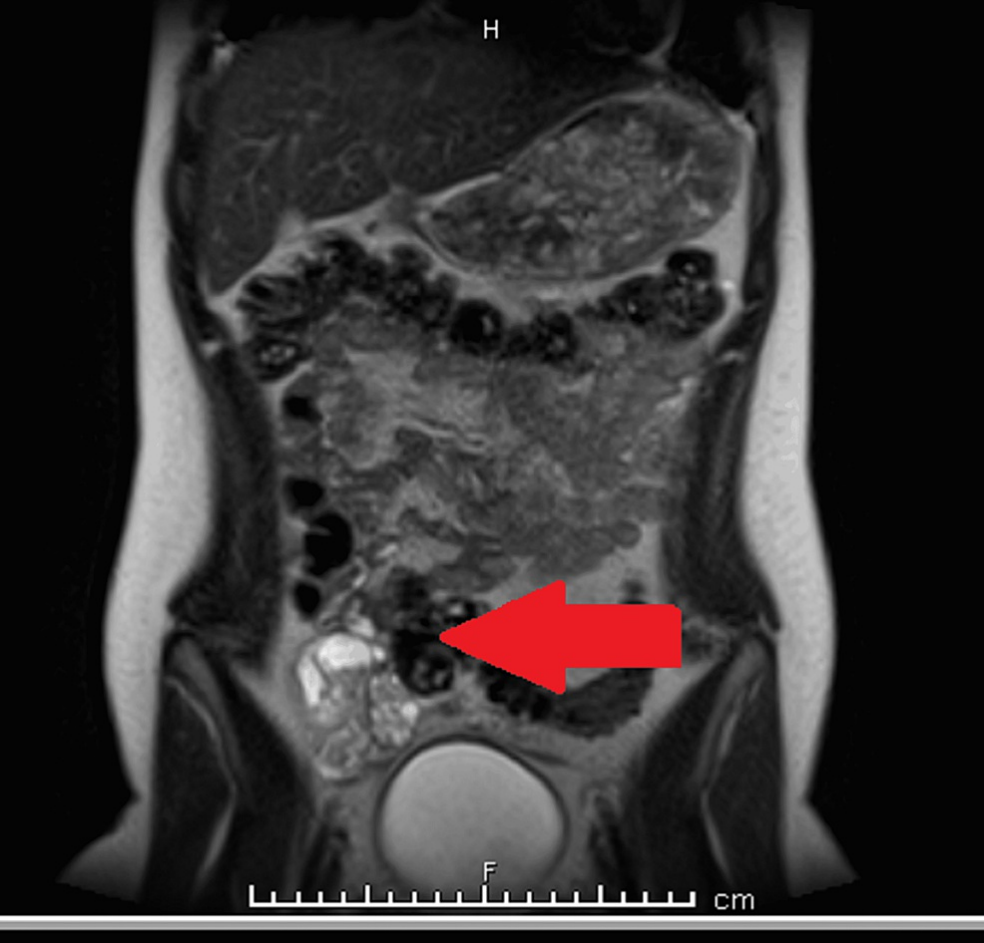
MRI showing right salpingitis

Figure 5 shows a follow-up MRI one week after discharge revealing dilatation of the right fallopian tube with enhancement and mild surrounding inflammatory changes consistent with active inflammatory pelvic disease.

Compared to a prior MRI eight months previous which was also done during acute illness, the inflammatory process was improved. No tubo-ovarian abscess was identified. The ovaries were unremarkable. No ectopic ureter was identified.

The patient followed up with her PMD a few weeks after discharge at which time she complained again of right lower quadrant pain. The primary medical doctor (PMD) verified no sexual activity and obtained a repeat ultrasound which showed hydrometra and hydrocolpos as well as a right-sided hemorrhagic cyst. No hydrosalpinx was seen. The PMD prescribed nitrofurantoin for three weeks to avoid infected urine refluxing into her uterus and referred the patient to pediatric urology. At this time, the patient’s mother offered additional history that her daughter holds her urine while playing sports and has “dribbled” urine for years. Of note, the patient was seen six years prior by a pediatric urologist for a history of recurrent UTIs and diagnosed with a spinning top urethra (STU) on voiding cystourethrography (VCUG). An ultrasound at that time showed normal kidneys but a fairly large post void residual. On revisiting the case with pediatric urology, she was felt to have multifactorial voiding dysfunction characterized by constipation, retention of urine, STU, and recurrent UTIs.

As of the writing of this case study, our patient has been two years free from any further episodes of salpingitis and UTI. Of note, she stopped playing sports, something she had been doing at a competitive level for several years. In addition, she was started on an oral contraceptive pill (OCP) by her gynecologist for dysmenorrhea. PMD reported improved hygiene attributed to maturity.

## Discussion

An STU is non-obstructive urethral dilatation seen on VCUG. It has been proposed as a consequence of increased posterior urethral pressure from unstable bladder contractions resisted by voluntary increase in distal sphincter tension to prevent urinary leakage. Congenital wide bladder neck anomaly may contribute to these cases [2]. Eighty-four children with STU were compared to 70 children with dysfunctional voiding and normal urethral width in a retrospective study on neurologically intact children with dysfunctional voiding. STU was more common in females and related to recurrent UTIs, VUR, poor bladder compliance, and an increase in voiding pressure [3].

Salpingitis or pyosalpinx refers to infection and associated inflammation in the fallopian tubes. Its presentation mimics that of acute appendicitis. Although not synonymous, it is often referenced with respect to pelvic inflammatory disease (PID) as it can be a complication. In sexually active females, it is most commonly caused by *Chlamydia trachomatis* and *Neisseria gonorrhoeae* [4]. Due to this, sexual abuse should be considered in young females with PID, although there are no specific instances of this reported in the literature. There have been a limited number of cases published on severe gynecological infections in precoital females.

A review of eight cases of PID in non-sexually active pediatric-adolescent females (ages: 11-16 years of age) showed over half had evidence of ascending infections with four cases associated with *E. coli* and one case from *Streptococcus viridans* [5]. A case report that highlighted eight prior case reports of tubo-ovarian abscesses (TOA) in virginal adolescent females also reported a predominant number of *E. coli* cases, although there were a number of overlapping cases in these two reports [5]. On review of these nine TOA cases, a number of children were noted to be obese. Obesity may have increased vulvar adiposity, which potentially can lead to a recessed urethral meatus, and/or chronic pooling of urine in the posterior vagina [6]. This would provide a mechanism for urinary pathogens to ascend into the upper genital tract. 

In a recent case series of an additional 10 cases of TOA, only one case was reported to have a recurrence. In comparison to 33 previously published cases these authors reviewed, inclusive of cases reviewed above, these 10 cases generally were culture negative due to empiric antibiotic use in six of the 10 patients. Seven of the 10 patients were treated with pelvic washout, four as a primary treatment modality, and three after failing antibiotic therapy. In their review, the majority of patients required surgery within the first three days of diagnosis [7].

In the various case series reviewed above, ascending infection was noted as a potential cause in many of these reports, but documentation of thorough urodynamic studies was lacking. Chronic constipation has also been associated with recurrent UTIs and has been proposed to be related to these cases [8]. Our patient did have a remote history of this and is described to have soiling of her underwear in the past. 

There were a number of associations in a small number of patients, such as complication of inflammatory bowel disease (IBD) with *Bacteroides uniformis*, coagulase-negative staphylococcus, and *Streptococcus milleri* identified on cultures, immune suppression second to renal transplant, VACTERL, and a case with history of imperforate anus with didelphys [7]. Underlying immunodeficiencies, fistulas with IBD, and bowel translocation from surgery or obstruction have all also been proposed contributors [9]. 

With respect to our patient, we believe that her STU contributed to the presence of aseptic urine in the vagina, which then served as a nidus for bacterial growth, and ascending infection in the genitourinary tract leading to pyosalpinx. Gynecology indicated that the patient had a dilated cervical os, which could contribute to this phenomenon. Urology confirmed the presence of an STU, a known cause of retained vaginal urine as well as recurrent UTIs [10]. It is important to note that our patient experienced complete resolution of her symptoms between episodes, suggesting her infection was adequately treated each time. Caregivers should be aware of this type of presentation as it can occur indolently, without fever as in our patient’s first initial presentation. This is particularly important since infertility and an increased chance of ectopic pregnancy are potential long-term sequelae of salpingitis.

In summary, precoital females can acquire PID with presentation commonly mimicking appendicitis. In a small number of published cases, PID in precoital females has been linked to IBD, obesity, constipation, recurrent UTIs, and poor hygiene [11]. Our case report now documents the role of an STU in the development of pyosalpinx in a precoital female and supports a thorough evaluation for contributing anatomic irregularities in these cases, particularly if they recur. 

## Conclusions

In summary, precoital females can acquire PID with presentation commonly mimicking appendicitis. In a small number of published cases, PID in precoital females has been linked to IBD, obesity, constipation, recurrent UTIs, and poor hygiene. Our case report now documents the role of an STU in the development of pyosalpinx in a precoital female and supports a thorough evaluation for contributing anatomic irregularities in these cases, particularly if they recur. 
